# Diverticulitis complicated by colovenous fistula formation and pylephlebitis

**DOI:** 10.1093/jscr/rjab591

**Published:** 2022-01-14

**Authors:** Ada Maria Krzak, Adam Townson, Yogeshkumar Malam, John Mathews

**Affiliations:** Department of Surgery, Hinchingbrooke Hospital, Huntingdon, UK; Department of Surgery, Hinchingbrooke Hospital, Huntingdon, UK; Department of Surgery, Hinchingbrooke Hospital, Huntingdon, UK; Department of Surgery, Hinchingbrooke Hospital, Huntingdon, UK

## Abstract

Acute diverticulitis is associated with a range of complications including fistula formation. Colovenous fistula formation, where there is a fistula between the inferior mesenteric vein and colon, is an extremely rare and serious complication of diverticulitis. Pylephlebitis, which is defined as infective suppurative thrombosis of the portal vein, is another uncommon complication of any intra-abdominal source of infection, including diverticulitis. Both complications are independently associated with significant morbidity and mortality. We report a case of a patient with acute diverticulitis who subsequently developed both colo-venous fistula and pylephlebitis and was successfully managed conservatively.

## INTRODUCTION

Acute diverticulitis is associated with a spectrum of complications, including intestinal obstruction, perforation, bleeding, abscess formation and fistulation among others [1]. Fistula formation is observed in 4–20% of cases. In terms of pathophysiology, fistulation is understood to occur when a diverticular abscess ruptures or extend into an adjacent organ. The most frequent fistulas are (in descending order): colovesical, colovaginal, coloenteric, colouterine and colocutaneous. The formation of a colovenous fistula, however, is an extremely rare complication of diverticulitis, associated with poor outcomes and mandating surgery.

We report a case of a patient with acute diverticulitis complicated by concurrent colovenous fistula formation and pylephlebitis, of which very few cases have previously been reported. To our knowledge, our case is the only one in which the patient was successfully managed conservatively.

## CASE REPORT

A 68-year-old woman presented to the emergency department with fever, new-onset confusion, increasing shortness of breath and a mild cough. She also reported 3 days of diarrhoea, which had settled prior to admission. She denied any other symptoms, including abdominalpain.

Past medical history included chronic obstructive pulmonary disease, type 2 diabetes mellitus and congestive cardiac failure (with a left ventricular ejection fraction of <25%). The patient was previously diagnosed with diverticular disease with previous episodes of diverticulitis.

On examination, the patient was septic with tachycardia, hypotension, pyrexia and had a new increased oxygen requirement, saturating 94% on 4L oxygen via nasal cannula. Chest auscultation revealed left basal crepitations. Abdominal examination was unremarkable.

Blood tests revealed raised inflammatory markers (WCC 11.6 × 10^9^/l; CRP 271 mg/l) and a raised d-dimer (1765 ng/ml). Liver function, renal function and electrolytes were within normal limits. A computed-tomography pulmonary-angiogram on admission was negative for pulmonary embolism or any focal consolidation. She was admitted under the medical team and treated with antibiotics for sepsis of unknown source.

Despite antibiotic treatment, the patient had persistently spiking fevers and complained of new-onset abdominal pain and diarrhoea. On examination, her abdomen was soft with moderate tenderness in the left iliac fossa. There were no signs of peritonism. Blood tests showed rising inflammatory markers and worsening renal function with the new development of stage 2 acute kidney injury. Blood cultures grew vancomycin-resistant enterococci.

**Figure 1:**
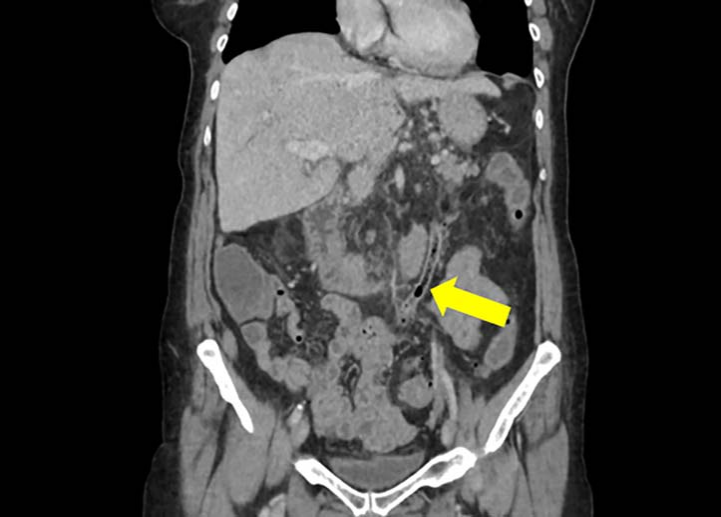
Coronal section of non-contrast CT scan demonstrating the colovenous fistula between the inferior mesenteric vein and sigmoid colon (yellow arrow); air is seen tracking within the lumen of the inferior mesentericvein.

**Figure 2:**
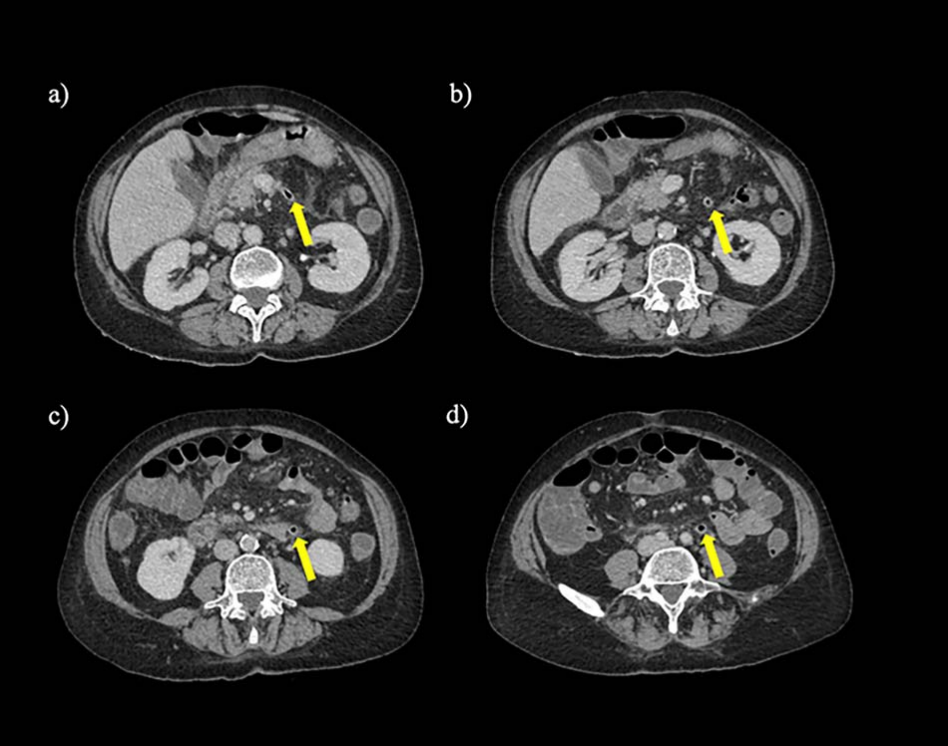
Axial section of a non-contrast CT scan with showing air tracking within the inferior mesenteric vein at various levels (yellow arrow).

**Figure 3:**
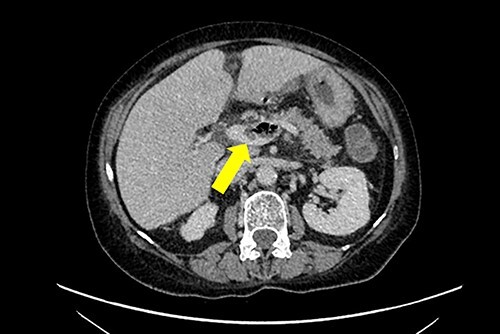
Axial section of a non-contrast CT scan with yellow arrow showing portal vein thrombosis with extension to the left intrahepatic portalvein.

A non-contrast-enhanced computed tomography (CT) scan of the abdomen and pelvis was performed. This demonstrated acute sigmoid diverticulitis with air tracking along the course of the inferior mesenteric vein (Figs 1 and 2). Additionally, a large portal vein thrombus was seen with extension to the left intrahepatic portal vein (Fig. 3) and evidence of portal venousgas.

Therefore, the patient was diagnosed with acute diverticulitis complicated by the presence of colovenous fistula involving the inferior mesenteric vein and sigmoid colon.

The patient was commenced on anticoagulation with therapeutic dose dalteparin and continued antibiotic treatment with tazocin and linezolid.

The patient subsequently clinically deteriorated and developed septic shock. She was transferred to the intensive care unit, requiring vasopressor support. Given the patient’s comorbidities and poor performance status, it was deemed that she would be extremely high risk for surgery and a conservative approach was followed.

The patient eventually responded to conservative treatment and improved clinically with resolution of her symptoms such as fever and improvement of inflammatory markers and renal function. She was able to be discharged home, 7 weeks after admission, with dalteparin converted to apixaban on discharge.

At follow-up 4 weeks following discharge, she remained clinically well with no recurrence of symptoms. Interval CT imaging 4 weeks following her discharge demonstrated that the colovenous fistula had sealed off with minimal changes to the portal vein thrombosis.

## DISCUSSION

We describe a case of acute diverticulitis complicated by the presence of a colo-venous fistula involving the inferior mesenteric vein, which is an extremely rare complication with only 9 previously reported cases in the literature (Table 1).

**Table 1:** Previous reported cases of colovenous fistula secondary to acute colonic diverticulitis and their key clinical features

**Author (Year)**	**Patient**	**Diagnosis**	**Blood culture**	**Pylephlebitis (vessel affected)**	**Management**	**Outcome**
Sonnenshein (1986) [3]	73 M	Barium enema	*Escherichia coli*	No	Laparotomy + Hartmann’s procedure	Died
Sonnenshein (1986) [3]	73 M	Gastrografin enema	*Escherichia coli, Enterococcus spp., Bacteroides fragilis*, *Bacteroides ovatus, Bacteroides distasonis*, *Pseudomona aeruginosa, Clostridium Perfringens*	No	Laparotomy + Hartmann’s procedure	Died
Rossmann (1997) [2]	32 M	CT and barium enema	*-*	No	Laparotomy + Hartmann’s procedure	Died
Heye (2002) [5]	49 M	CT and gastrografin enema	*-*	Yes (Portal vein)	Laparotomy + Hartmann’s procedure	Survived
Strålin (2006) [8]	67 F	CT	*Bacteroides fragilis, Enterococcus faecalis*, *Lactobacillus spp., Candida albicans, Candida dubliniensis,* Coagulase Negative *Staphylococci spp.*	No	Laparotomy + Hartmann’s procedure	Survived
Strålin (2006) [8]	53 M	CT + contrast enema	*Escherichia coli, Bacteroides fragilis, Candida dubliniensis*	No	Laparotomy + Hartmann’s procedure	Survived
Radzina (2010) [6]	46 M	CT	*Escherichia coli and Enterococcus spp.*	Yes (Portal vein)	Multiple laparotomies –unspecified	Survived
Yates (2018) [7]	54 M	CT	*Escherichia coli*	Yes (Right posterior branch of portal vein)	Laparotomy + Hartmann’s procedure	Survived
Loobuyck (2021) [4]	59 M	PET-CT	*Escherichia coli*	No	Laparoscopic sigmoidectomy with end-to-end anastomosis	Survived

M = Male; F = Female; PET-CT = Positron emission tomography-computed tomography.

Historically, contrast enema was the diagnostic test of choice, with intravasation of contrast media used to visualize the fistula [2, 3]. This has been superseded with modern contrast-enhanced CT scanning with the demonstratable finding of air seen in the inferior mesenteric vein [4].

The formation of colovenous fistula is a serious complication of diverticulitis, establishing a direct path for the translocation of colonic bacteria into the venous system. This may result in pylephlebitis, which is defined as the infective suppurative thrombosis of the portal vein or one of its branches. Of the previous reported cases of colovenous fistula, evidence of pylephlebitis was reported in three patients [5–7], with our case adding a further one to these.

In all cases reported in the literature, the management of colovenous fistula involved surgery with a resection of the affected segment of colon. However, this is associated with high morbidity and mortality [2, 3]. Our patient was unfit to have surgery given her co-morbidities and overall general condition; she was managed conservatively with a prolonged course of intravenous antibiotics and therapeutic anticoagulation.

Given the rarity of colovenous fistulas, there is a paucity of evidence guiding their management. Though surgical resection may be performed, our case demonstrates that conservative management of colovenous fistulas may work in selected cases, especially in surgically high-risk patients, and may be considered a feasible treatment option.
